# Commentary: enhancing real-world applicability of MRI-based prediction models for axillary pCR in breast cancer

**DOI:** 10.1097/JS9.0000000000003275

**Published:** 2025-08-22

**Authors:** Tujin Wu, Bin Chen, Xiaozhen Zhan

**Affiliations:** Department of Thyroid and Breast Surgery, Xiamen Humanity Hospital, Fujian Medical University, Xiamen, China

*Dear Editor,*

We read with great interest the recent article by Liu *et al* entitled “*Integrating clinical-pathological-MRI features to predict axillary pathological complete response in early breast cancer*^[1]^,” published in the *International Journal of Surgery*. This study developed a clinical-pathological-MRI-based model to predict axillary pathological complete response (apCR) following neoadjuvant therapy (NAT), offering valuable insights into noninvasive axillary management. However, several key aspects of study design and model development warrant further discussion to contextualize the findings and improve future clinical applicability.

First, the study may be subject to selection bias. Inclusion criteria required patients to undergo serial MRI and pathological confirmation after surgery, likely excluding those who did not undergo MRI or were managed with sentinel lymph node biopsy alone. As such patients represent a substantial portion of real-world breast cancer cases, their exclusion may reduce model generalizability. To enhance inclusivity, future studies could incorporate these patients via missing data imputation or develop stratified prediction models that address patients with and without MRI availability separately.

Second, treatment heterogeneity over time was not explicitly addressed. Although NAT regimens were reportedly standardized according to clinical guidelines, the study did not clarify whether inter-patient variations (e.g., treatment adjustments due to adverse events^[2]^) or evolving standards^[3]^ (e.g., increasing use of genomic or immunologic stratification^[4]^) were considered. Such variability may confound axillary response and affect model performance across settings.

Third, key demographic and social factors were underutilized. Variables like age, body mass index, and menopausal status were reported but not incorporated into the model or analyzed in subgroups, despite prior studies showing their relevance to NAT response^[5,6]^. Additionally, if the cohort underrepresents older adults, patients with comorbidities, or those from underserved backgrounds, concerns may arise regarding fairness and external applicability. We suggest future multicenter validations using demographically diverse cohorts to assess equity and model robustness.

Finally, the clinical implementation pathway remains unclear. Although the model demonstrated good predictive performance, it lacked defined thresholds for clinical decision-making and provided no actionable recommendations (e.g., on whether ALND could be omitted). Without explicit clinical integration strategies, its application in real-world workflows may be limited.

In conclusion, Liu *et al* have introduced a promising model for apCR prediction, and their work contributes meaningfully to the advancement of personalized breast cancer care. Addressing the methodological issues outlined above will enhance the model’s generalizability, clinical relevance, and potential for integration into multidisciplinary decision-making.

## Data Availability

All data analyzed or generated during this study are included in the article.

## References

[1] Shang JM Chen JZ Gao XD. Integrating clinical-pathological-MRI features to construct a prediction model for pathological complete remission of axillary lymph nodes after neoadjuvant therapy: a retrospective study. Int J Surg 2025. doi:10.1097/JS9.0000000000003070. PMC12626574 40694024

[2] Yılmaz C Zengel B Üreyen O. A comprehensive analysis of neoadjuvant chemotherapy in breast cancer: adverse events, clinical response rates, and surgical and pathological outcomes—Bozyaka experience. Cancers (Basel) 2025;17:163. 39857945 10.3390/cancers17020163 PMC11763700

[3] Spring LM Bar Y Isakoff SJ. The evolving role of neoadjuvant therapy for operable breast cancer. J Natl Compr Canc Netw 2022;20:723–34. 35714678 10.6004/jnccn.2022.7016

[4] Heater NK Warrior S Lu J. Current and future immunotherapy for breast cancer. J Hematol Oncol 2024;17:131. 39722028 10.1186/s13045-024-01649-z PMC11670461

[5] Boughey JC Hoskin TL Goetz MP. Neoadjuvant chemotherapy and nodal response rates in luminal breast cancer: effects of age and tumor Ki67. Ann Surg Oncol 2022;29:5747–56. 35569077 10.1245/s10434-022-11871-z PMC9466990

[6] Holm JB Skovbjerg SB Nielsen HM. The association between body mass index and neoadjuvant chemotherapy response in patients with breast cancer. Breast Cancer Res 2025;27:130. 40646617 10.1186/s13058-025-02083-w PMC12254981

[7] Agha RA Mathew G Rashid R. Transparency in the reporting of Artificial INtelligence – the TITAN guideline. Prem J Sci 2025;10:100082.

